# Accounting for Expected Adjusted Effect

**DOI:** 10.3389/fpsyg.2020.542082

**Published:** 2020-09-17

**Authors:** Kimmo Sorjonen, Bo Melin, Michael Ingre

**Affiliations:** ^1^Department of Clinical Neuroscience, Karolinska Institutet, Stockholm, Sweden; ^2^Department of Psychology, Faculty of Social Sciences, Stockholm University, Stockholm, Sweden; ^3^Institute for Globally Distributed Open Research and Education (IGDORE), Stockholm, Sweden

**Keywords:** adjustment, confounder, expected effect, regression analysis, reliability, simulation, type 1-error

## Abstract

The point that adjustment for confounders do not always guarantee protection against spurious findings and type 1-errors has been made before. The present simulation study indicates that for traditional regression methods, this risk is accentuated by a large sample size, low reliability in the measurement of the confounder, and high reliability in the measurement of the predictor and the outcome. However, this risk might be attenuated by calculating the expected adjusted effect, or the required reliability in the measurement of the possible confounder, with equations presented in the present paper.

## Introduction

To analyze the regression effect of a predictor on an outcome while adjusting for possible confounders is very common in non-experimental research. However, there are some indications that adjustment for confounders do not always guarantee protection against spurious findings. For example, the point that it can be futile to control for underlying confounders that are measured with low reliability has been made on numerous occasions (e.g., Stouffer, 1936; Kahneman, 1965; Greenland, 1980; Phillips and Davey Smith, 1992; Brenner, 1993; Fewell et al., 2007; Brunner and Austin, 2009; Shear and Zumbo, 2013; Lee and Burstyn, 2016; Westfall and Yarkoni, 2016; Pei et al., 2019).

Kahneman (1965), for example, presents the equation below, which gives the partial correlation between X and Y when adjusting for Z, taking the reliability in the measurement of Z (*r^2^_ZZ_*) into account. We see that even if X and Y would have strong correlations with the true value on Z (*R*_*XZ*_ and *R*_*YZ*_, respectively), if the reliability in the measurement of Z is low, the estimated partial correlation will be close to the zero-order correlation (*r*_*XY*_) and the adjustment does not have anticipated effect.

(1) $$r_{X\,Y.Z}=\frac{r_{X\,Y}-r_{Z\,Z}^{2}\,R_{X\,Z}\,R_{Y\,Z}}{\sqrt{1-r_{Z\,Z}^{2}\,R_{X\,Z}^{2}}\,\sqrt{1-r_{Z\,Z}^{2}\,R_{Y\,Z}^{2}}}$$

The objective of the present simulation study was to investigate and demonstrate how the effect of a predictor on an outcome while adjusting for a confounder is affected by the reliability in the measurement of the confounder, as well as the reliability in the measurement of the predictor and the outcome variable, sample size, and size of the true independent association between predictor and outcome. We will also evaluate a method for accounting for expected (spurious) adjusted effects of the predictor on the outcome.

## Method

**FIGURE 1:**
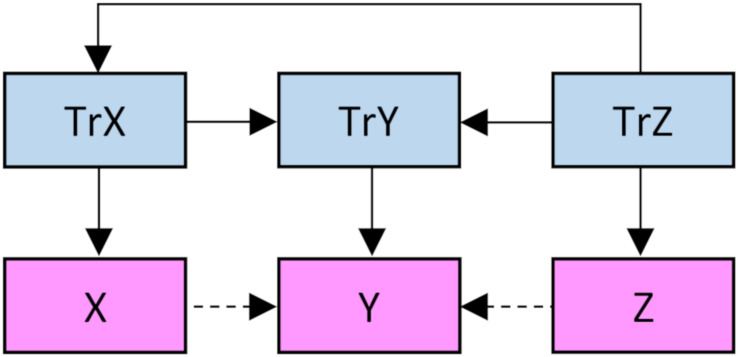
Illustration of the present simulation, with various degrees of confounding effects of true Z on true X/Y, various degrees of true adjusted effects of true X on true Y, and various degrees of reliability in the measurement of Z/X/Y. The main outcome is the effect of observed X on observed Y while adjusting for observed Z.

Using R 4.0.2 statistical software (R Core Team, 2020) and the MASS package (Venables and Ripley, 2002), in a first set of simulations, data was simulated and analyzed through the following steps (Figure 1, script available at^1^): (1) 20, 100, 500, or 2500 virtual subjects were allocated a true *Z* value from a random standard normal distribution; (2) The subjects were allocated true X and true *Y* values from random standard normal distributions with defined population correlations (0.1, 0.35, 0.6, or 0.85) with the true Z distribution (same for both) and with a defined adjusted effect of true X on true Y (drawn from a random uniform distribution between 0 and 1); (3) The subjects were allocated observed Z, X, and Y scores from random standard normal distributions with defined population correlations (square root of the defined reliability, which was set to 0.8 for all three variables, and consequently the correlation was set to 0.894) with their respective true scores; and (4) The effect of observed X on observed Y while adjusting for observed Z was analyzed with ordinary least squares linear regression. As all variables were standardized, the effects correspond to standardized beta weights. We ran 1000 simulations for each of the 16 combinations of sample size and defined population correlation between true Z and true X/Y, i.e., 16,000 simulations in total. In a second set of simulations, the sample size was fixed at 500 and the population correlation between true Z and true X/Y at 0.5 while we used 0.4, 0.6, 0.8, and 0.99 (the calculations did not converge if using the integer 1) as values for the reliability in the measurement of Z and the reliability in the measurement of X/Y (same for both). Again, we ran 1000 simulations for each of the 16 combinations of these reliabilities.

The standardized regression effect of a predictor X on an outcome Y while adjusting for a potential confounder Z is given by the equation (Cohen et al., 2003):

(2) $$\beta_{X\,Y.Z}=\frac{r_{X\,Y}-r_{X\,Z}\,r_{Y\,Z}}{1-r_{X\,Z}^{2}}$$

As the correlations between observed Z, X, and Y in the present simulation equals the product of their correlations with true scores, and the associations between these true scores (see Figure 1), it can be shown (see Appendix) that the expected adjusted standardized effect, in the case without any true independent association between X and Y, is given by:

(3) $$E\,\left|\beta_{X\,Y.Z}\right|=\frac{r_{X\,Z}\,r_{Y\,Z}\,\left(1-r_{Z\,Z}^{2}\right)}{r_{Z\,Z}^{2}\,\left(1-r_{X\,Z}^{2}\right)}$$

In Eq. 3 we see that with a decrease in the reliability of the measurement of Z (*r*^2^_*ZZ*_), we will get an increase in the numerator and a decrease in the denominator and, hence, a strengthening of the expected adjusted effect. This expected adjusted effect can be quite substantial even if the true adjusted effect of true X on true Y while adjusting for true Z is zero.

The significance of an adjusted (or an un-adjusted) regression effect is usually calculated by dividing the coefficient with its standard error, which gives a *T*-value, and then finding the corresponding *p*-value. The *p*-value stands for the estimated probability to get a regression coefficient that deviates as much (or more) from zero as the observed regression coefficient does, if the true regression coefficient in the population actually is zero. If this *T*-value is significant (commonly defined as *p* < 0.05) it is usually concluded that the adjusted effect differs from zero (hence the subscript below) and that there is an independent association between X and Y when adjusting for Z:

(4) $$T_{0}=\frac{\beta_{X\,Y.Z}}{S\,E\,\left(\beta_{X\,Y.Z}\right)}$$

Besides this traditional zero-order significance test, in the present simulation we also calculated the significance of the difference between the observed and the expected adjusted effect (AEAE), as calculated with Eq. 3. In this case a significant finding would be taken to indicate that the independent association between X and Y is stronger than can be expected due to purely spurious reasons:

(5) $$T_{A\,E\,A\,E}=\frac{\beta_{X\,Y.Z}-E\,\left|\beta_{X\,Y.Z}\right|}{S\,E\,\left(\beta_{X\,Y.Z}\right)}$$

Associations between the true adjusted effect (as defined in the simulations) and the probability for a significant observed adjusted effect as given by the zero-order significance test (Eq. 4) as well as the test accounting for expected adjusted effect (Eqs. 3 and 5) were analyzed with logistic regression analyses. In these analyses, the significance of the effect of observed X on observed Y while adjusting for observed Z (with 0 for *p* ≥ 0.05 and 1 for *p* < 0.05) was the binary outcome while the size of the true adjusted effect of true X on true Y while adjusting for true Z was the continuous predictor. Based on the results from these logistic analyses, the probability to get a significant (*p* < 0.05) observed adjusted effect of observed X on observed Y while adjusting for observed Z could be estimated for different degrees of true adjusted effect of true X on true Y while adjusting for true Z. These estimated probabilities could vary between 0 (meaning that the observed adjusted effect was predicted to never become significant) and 1 (meaning that the observed adjusted effect was predicted to always become significant). If the true adjusted effect of true X on true Y while adjusting for true Z equals zero (as defined by us in the simulation), this estimated probability stands for the risk to conduct a type 1-error, i.e., to conclude that there is an independent association between X and Y while adjusting for Z when there actually is not. If, on the other hand, the true adjusted effect is not zero (as defined by us in the simulation), this estimated probability stands for power, i.e., the ability to reveal an independent association between X and Y that actually exists.

## Results

### Effects of Degree of Confounding and Sample Size

**FIGURE 2:**
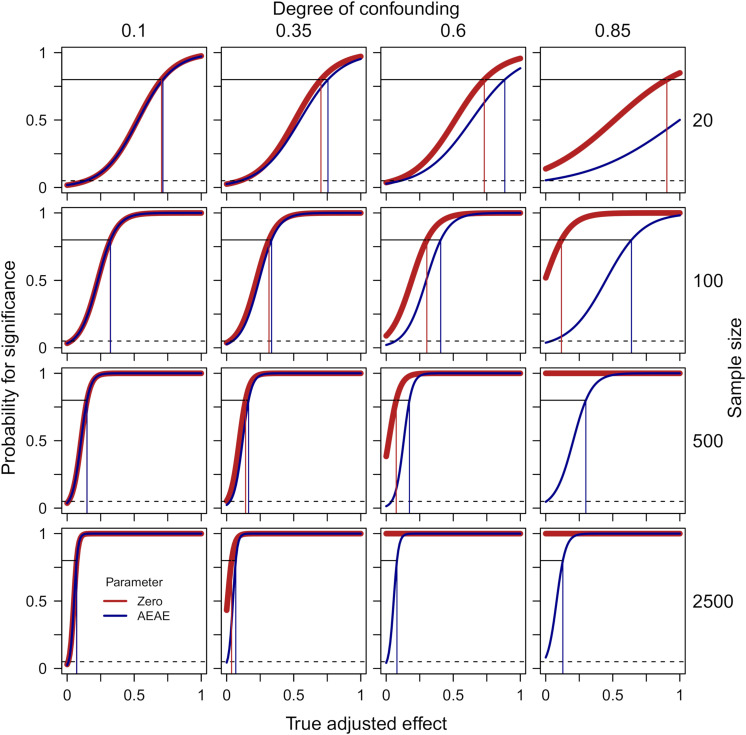
Probabilities for a significant finding from zero-order significance tests (Eq. 4) of the effect of X on Y while adjusting for Z (thick red line) as well as when accounting for the expected adjusted effect (AEAE-test, Eqs. 3 and 5, dark blue line) as functions of the true adjusted effect, separately for four degrees of confounding (i.e., correlation between true Z and true X/Y, columns) and four sample sizes (rows). The dashed lines show *p* = 0.05. The thin vertical lines indicate required degree of true adjusted effect for power = 0.80 for the zero-order significance test (red) and the AEAE-test (dark blue), respectively. The reliability in measurement of X/Y/Z was fixed at 0.8 in these simulations.

Figure 2 presents probabilities for a significant finding from zero-order significance tests (Eq. 4) of the effect of X on Y while adjusting for Z (thick red line) as well as when accounting for the expected adjusted effect (the AEAE-test, Eqs. 3 and 5, dark blue line) as functions of the true adjusted effect (defined in the simulation), separately for four degrees of confounding (i.e., correlation between true Z and true X/Y) and four sample sizes. The following can be noted: (1) With a low degree of confounding (column 1), the two methods give identical results; (2) With a high degree of confounding (columns 4 and, to a lesser degree, 3), the zero-order significance test exhibits a high risk for type 1-error when the true adjusted effect = 0, and this risk is accentuated by a large sample size; (3) The AEAE-test provides good protection against type 1-errors irrespective of degree of confounding and sample size (the probability for a significant result when the true adjusted effect = 0 is always close to the nominal 5%); and (4) This extra protection against type 1-errors comes with some decrease in power when there is a high degree of confounding and when the true adjusted effect is relatively weak (there is a gap between the red and the blue line in the left part of the panels in column 4). For example, when *N* = 100 and degree of confounding = 0.85, the estimated degree of true adjusted effect required for power = 0.80 (risk for type 1-error if true adjusted effect = 0) is 0.117 (0.519) and 0.639 (0.038) for the zero-order significance test and the AEAE-test, respectively.

### Effect of Reliability

**FIGURE 3:**
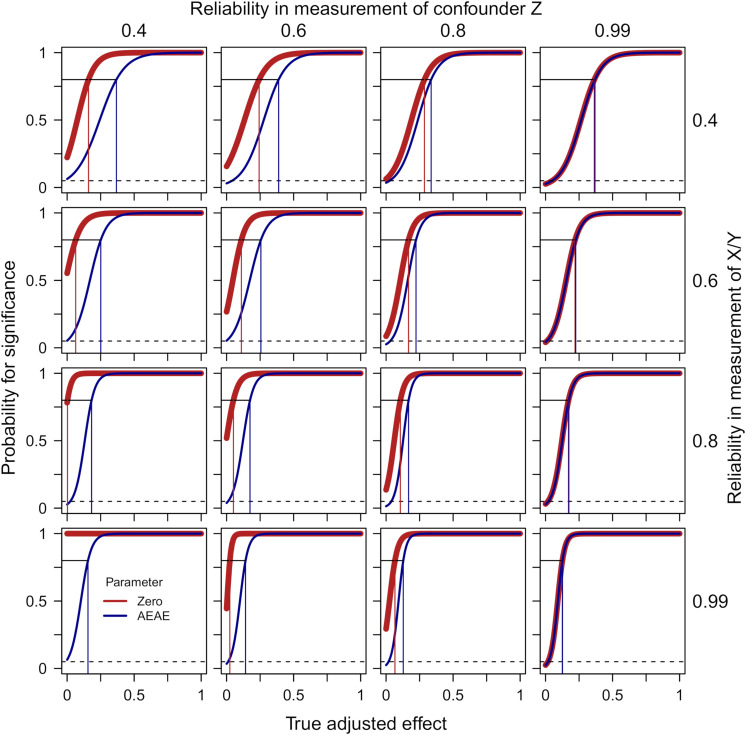
Probabilities for a significant finding from zero-order significance tests (Eq. 4) of the effect of X on Y while adjusting for Z (thick red line) as well as when accounting for the expected adjusted effect (AEAE-test, Eqs. 3 and 5, dark blue line) as functions of the true adjusted effect, separately for four degrees of reliability in the measurement of Z (columns) and four degrees of reliability in the measurement of X/Y (rows). The dashed lines show *p* = 0.05. The thin vertical lines indicate required degree of true adjusted effect for power = 0.80 for the zero-order significance test (red) and the AEAE-test (dark blue), respectively. The sample size was fixed at 500 and the degree of confounding at 0.5 in these simulations.

Figure 3 presents probabilities for a significant finding from zero-order significance tests (Eq. 4) of the effect of X on Y while adjusting for Z (thick red line) as well as when accounting for the expected adjusted effect (the AEAE-test, Eqs. 3 and 5, dark blue line) as functions of the true adjusted effect (defined in the simulation), separately for four degrees of reliability in the measurement of the confounder Z and four degrees of reliability in the measurement of predictor X and outcome Y. The following can be noted: (1) With a near-perfect reliability in the measurement of Z (column 4), the two methods give identical results; (2) With a low reliability in the measurement of Z (columns 1 and, to a lesser degree, 2), the zero-order significance test exhibits a high risk for type 1-error when the true adjusted effect = 0, and this risk is accentuated by a high reliability in the measurement of X/Y; (3) The AEAE-test provides good protection against type 1-errors irrespective of reliability in the measurement of Z and reliability in the measurement of X/Y (the probability for a significant result when the true adjusted effect = 0 is always close to the nominal 5%); and (4) This extra protection against type 1-errors comes with some decrease in power when Z is measured with low reliability and when the true adjusted effect is relatively weak (there is a gap between the red and the blue line in the left part of the panels in column 1). For example, when the reliability in the measurement of X/Y = 0.8 and the reliability in the measurement of *Z* = 0.4, the estimated degree of true adjusted effect required for power = 0.80 (risk for type 1-error if true adjusted effect = 0) is 0.003 (0.782) and 0.182 (0.027) for the zero-order significance test and the AEAE-test, respectively.

## Discussion

The present simulation indicates that with some degree of true confounding from a factor Z, a relatively strong, and often significant, but spurious adjusted effect of a predictor X on an outcome Y can be expected even if the true adjusted effect equals zero. The risk for such spurious findings is accentuated by a large sample size, low reliability in the measurement of Z, and high reliability in the measurement of X and Y. Hence, if the effect of X on Y remains significant when adjusting for Z, this can in many situations not be interpreted as a strong indication of a true independent association.

One way to decrease the risk for spurious findings and type 1-errors would be to use multiple indicators of the variables of interest and structural equation modeling (Westfall and Yarkoni, 2016). However, if multiple indicators are not available, one could calculate the size of the expected adjusted effect with the presented Eq. 3 and see if the found effect differs significantly from this expected effect (Eq. 5) rather than being content with a significant deviation from zero. This would require an estimate of the reliability in the measurement of the potential confounder Z, something that could be based on calculations of homogeneity, test-retest correlations etc. Replacing the expected effect in Eq. 3, i.e., the left-hand side, with the lower limit (closest to zero) of the confidence interval of the observed adjusted effect (β_*LL*_), one could also calculate the degree of reliability in the measurement of Z that is required for the observed adjusted effect to be significantly stronger than the expected adjusted effect:

(6) $$r\,e\,q\,u\,i\,r\,e\,d\,r_{Z\,Z}^{2}=\frac{r_{X\,Z}\,r_{Y\,Z}}{\beta_{L\,L}\,\left(1-r_{X\,Z}^{2}\right)+r_{X\,Z}\,r_{Y\,Z}}$$

If the calculated required reliability given by Eq. 6 is unrealistically high one should be reluctant to conclude that there is an independent association between true X and true Y.

As an example, based on the adjusted effects presented in Table 1, Uchmanowicz and Gobbens (2015) concluded that both frailty and depression have independent associations, adjusting for each other, with physical and mental aspects of health-related quality of life among elderly patients with heart failure. In Table 1 we also present expected adjusted effects, as calculated with Eq. 3, for three possible degrees of reliability in the measurement of the confounder Z, namely 0.7, 0.8, and 0.9. Of course, if one has access to the data, the degree of reliability can be calculated through calculations of homogeneity (e.g., Cronbach’s alpha or McDonald’s omega) or possibly through calculations of test-retest correlations. We see in Table 1 that in many cases the expected adjusted effect is stronger than the lower limit of the confidence interval of the observed effect (β_*LL*_). In these cases, one should be reluctant to claim any non-spurious independent association between X and Y while adjusting for Z. If using Eq. 6, we see that the required reliability in the measurement of the other variable needs to be fairly high (0.72–0.85) for the observed adjusted effect to be significantly stronger than the expected adjusted effect (under the null hypothesis of no true independent association). It may very well be the case that Uchmanowicz and Gobbens’ measures had this required degree of reliability, but the degree of significance would be lower than if comparing, unrealistically, with an expected effect of zero.

**TABLE 1:** Example of findings from Uchmanowicz and Gobbens (2015) that would require a fairly high reliability in the measurement of the possible confounder (Z) in order for the adjusted effect of X on Y to be significantly stronger than the expected effect.

Y	PQoL	PQoL	MQoL	MQoL
X	Frailty	Depression	Frailty	Depression
Z	Depression	Frailty	Depression	Frailty
r_*XZ*_	0.66	0.66	0.66	0.66
r_*YZ*_	−0.61	−0.66	−0.74	−0.68
β_*XY.Z*_	−0.39	−0.33	−0.35	−0.49
SE_β_	0.10	0.10	0.09	0.09
β_*LL*_	−0.19	−0.13	−0.17	−0.31
E| β_*XY.Z*_|, r^2^_*ZZ*_ = 0.7	−0.31^*ns*^	−0.33^*ns*^	−0.37^*ns*^	−0.34^*ns*^
E| β_*XY.Z*_|, r^2^_*ZZ*_ = 0.8	−0.18	−0.19^*ns*^	−0.22^*ns*^	−0.20
E| β_*XY.Z*_|, r^2^_*ZZ*_ = 0.9	−0.08	−0.09	−0.10	−0.09
req. r^2^_*ZZ*_	0.79	0.85	0.83	0.72

*β_*XY.Z*_, observed effect of X on Y adjusted for Z; SE_β_, standard error of the observed adjusted effect; β_*LL*_, lower limit of the 95% CI of the observed adjusted effect; E| β_*XY.Z*_|, expected adjusted effect as given by Eq. 3; req. r^2^_*ZZ*_, required reliability in the measurement of Z for the observed adjusted effect to be significantly stronger than the expected effect; PQoL, physical quality of life; MQoL, mental quality of life; ^*ns*^ the observed adjusted effect does not differ significantly from the expected adjusted effect.*

Somebody might be alarmed by the indicated decrease in power when there is a high degree of true confounding, or a low reliability in the measurement of the confounder, in combination with a relatively weak true adjusted effect for the test accounting for expected adjusted effect (AEAE-test) compared to a traditional zero-order significance test, and think that this speaks against using the former method. Some kind of middle road might be to calculate both the zero-order and the AEAE-significance. If both of them are significant or non-significant the conclusion should be obvious (although important, we do not include issues of prior probabilities, p-hacking etc. into the present discussion). If the zero-order test is significant while the AEAE is not, on the other hand (the opposite should not happen), one should tread carefully, as this discrepancy could be indicative of a high degree of true confounding or a low reliability in the measurement of the confounder.

Some critique can be directed at the presented method for accounting for expected adjusted effect (the AEAE-test, Eqs. 3 and 5). For example, as the method was deduced from and then tested with the same algorithm for data generation (Figure 1), it might not be a big surprise that it seemed to perform quite well. We cannot be sure that the AEAE-test would perform equally well, i.e., with the same degree of protection against type 1-errors and power, had the data been generated in some other fashion, for example with several confounders that influence each other as well as the predictor and the outcome in an intricate network. However, neither can we be sure, actually we doubt it, that the traditional zero-order significance test of adjusted effects would perform any better in such situations.

Nonetheless, as the presented method (AEAE) is limited to a situation with one predictor and one possible confounder, while researchers often have to deal with several possible confounders, its practical usability is quite restricted. One could still, in a piecewise fashion, calculate the expected adjusted effect (Eq. 3) and the significance when accounting for this (Eq. 5), or the required reliability (Eq. 6), for each possible confounder separately, but it would of course be preferable if all of the confounders could be accounted for simultaneously. Maybe we, or somebody else, will be able to figure out such a method in the future. We would also like to add that just because the presented AEAE-method has limited practical usability, this does not mean that it is safe to continue using the zero-order significance test as usual. As the present study demonstrates, this method has a high risk of producing type 1-errors in certain situations.

## Conclusion

The present simulation indicates that with some degree of true confounding, there is a risk for adjustment for confounding through a traditional zero-order significance test to fail, resulting in type-1 errors. The risk is accentuated by a large sample size, low reliability in the measurement of the confounder, and high reliability in the measurement of the predictor and the outcome. We present an equation that can be used to calculate the size of the expected adjusted effect in a situation with no true independent association between the predictor and the outcome. To conclude that an adjusted effect is significant, this expected effect should not be included in the confidence interval of the observed adjusted effect. To the best of our knowledge this equation is novel, although Kahneman (1965) presents something similar for partial correlations (see Eq. 1 in the introduction). One difference is that our equation, contrary to Kahneman’s, does not include correlations involving true (unobserved) values on variables.

## Data Availability Statement

The datasets generated for this study are available on request to the corresponding author.

## Author Contributions

All authors conceived of the presented idea, discussed the results, proposed changes, contributed to the final manuscript, and approved the final version of the manuscript. KS carried out the simulations and analyses, and wrote an initial draft.

## Conflict of Interest

The authors declare that the research was conducted in the absence of any commercial or financial relationships that could be construed as a potential conflict of interest.

## Footnotes

1 https://osf.io/gxvfm/

## Appendix

See Figure 1 in the Method section. Assume that the true adjusted effect of X on Y equals zero; that *r_TrXTrZ_* = correlation between true X and true Z; *r_TrYTrZ_* = correlation between true Y and true Z; *r_XX_* = correlation between true X and observed X; *r_YY_* = correlation between true Y and observed Y; *r_ZZ_* = correlation between true Z and observed Z. The expected correlations between observed X, Y, and Z equals the following products:

*r_XY_* = *r_TrXTrZ_* × *r_TrYTrZ_* × *r_XX_* × *r_YY_* *e1* (the product of the four paths between observed X and Y)

*r_XZ_* = *r_TrXTrZ_* × *r_XX_* × *r_ZZ_* *e2* (the product of the three paths between observed X and Z)

*r_YZ_* = *r_TrYTrZ_* × *r_YY_* × *r_ZZ_* *e3* (the product of the three paths between observed Y and Z)

We can multiply the numerator and the denominator in the right part of Eq. 2 (see the Method section) by r^2^_*ZZ*_:

$\beta_{X\,Y.Z}=\frac{r_{Z\,Z}^{2}\times \left(r_{X\,Y}-r_{X\,Z}\times r_{Y\,Z}\right)}{r_{Z\,Z}^{2}\times \left(1-r_{X\,Z}^{2}\right)}=\frac{r_{Z\,Z}^{2}\times r_{X\,Y}-r_{Z\,Z}^{2}\times r_{X\,Z}\times r_{Y\,Z}}{r_{Z\,Z}^{2}\times \left(1-r_{X\,Z}^{2}\right)}$ *e4*

We can replace terms in the left part of the numerator in e4 with e1:

$r_{Z\,Z}^{2}\times r_{X\,Y}=r_{Z\,Z}^{2}\times r_{T\,r\,X\,T\,r\,Z}\times r_{T\,r\,Y\,T\,r\,Z}\times r_{X\,X}\times r_{Y\,Y}$ *e5*

From expressions e2 and e3 above we know that:

$r_{T\,r\,X\,T\,r\,Z}=\frac{r_{X\,Z}}{r_{X\,X}\times r_{Z\,Z}}$ *e6*

$r_{T\,r\,Y\,T\,r\,Z}=\frac{r_{Y\,Z}}{r_{Y\,Y}\times r_{Z\,Z}}$ *e7*

We can replace terms in e5 with e6 and e7 and simplify:

$r_{Z\,Z}^{2}\times r_{X\,Y}=\frac{r_{Z\,Z}^{2}\times r_{X\,Z}\times r_{Y\,Z}\times r_{X\,X}\times r_{Y\,Y}}{r_{X\,X}\times r_{Z\,Z}\times r_{Y\,Y}\times r_{Z\,Z}}=r_{X\,Z}\times r_{Y\,Z}$ *e8*

We can replace the left part of the numerator in e4 with e8 and simplify:

$\beta_{X\,Y.Z}=\frac{r_{X\,Z}\times r_{Y\,Z}-r_{Z\,Z}^{2}\times r_{X\,Z}\times r_{Y\,Z}}{r_{Z\,Z}^{2}\times \left(1-r_{X\,Z}^{2}\right)}=\frac{r_{X\,Z}\times r_{Y\,Z}\times \left(1-r_{Z\,Z}^{2}\right)}{r_{Z\,Z}^{2}\times \left(1-r_{X\,Z}^{2}\right)}$ *e9*

e9 is identical to Eq. 3 (see the Method section) and gives the expected effect of observed X on observed Y when adjusting for observed Z in a situation with no independent association between true X and true Y.
